# Procedures for Behavioral Experiments in Head-Fixed Mice

**DOI:** 10.1371/journal.pone.0088678

**Published:** 2014-02-10

**Authors:** Zengcai V. Guo, S. Andrew Hires, Nuo Li, Daniel H. O'Connor, Takaki Komiyama, Eran Ophir, Daniel Huber, Claudia Bonardi, Karin Morandell, Diego Gutnisky, Simon Peron, Ning-long Xu, James Cox, Karel Svoboda

**Affiliations:** Janelia Farm Research Campus, Howard Hughes Medical Institute, Ashburn, Virginia, United States of America; Department of Basic Neurosciences, University of Geneva, Geneva, Switzerland; Duke University Medical Center, United States of America

## Abstract

The mouse is an increasingly prominent model for the analysis of mammalian neuronal circuits. Neural circuits ultimately have to be probed during behaviors that engage the circuits. Linking circuit dynamics to behavior requires precise control of sensory stimuli and measurement of body movements. Head-fixation has been used for behavioral research, particularly in non-human primates, to facilitate precise stimulus control, behavioral monitoring and neural recording. However, choice-based, perceptual decision tasks by head-fixed mice have only recently been introduced. Training mice relies on motivating mice using water restriction. Here we describe procedures for head-fixation, water restriction and behavioral training for head-fixed mice, with a focus on active, whisker-based tactile behaviors. In these experiments mice had restricted access to water (typically 1 ml/day). After ten days of water restriction, body weight stabilized at approximately 80% of initial weight. At that point mice were trained to discriminate sensory stimuli using operant conditioning. Head-fixed mice reported stimuli by licking in go/no-go tasks and also using a forced choice paradigm using a dual lickport. In some cases mice learned to discriminate sensory stimuli in a few trials within the first behavioral session. Delay epochs lasting a second or more were used to separate sensation (e.g. tactile exploration) and action (i.e. licking). Mice performed a variety of perceptual decision tasks with high performance for hundreds of trials per behavioral session. Up to four months of continuous water restriction showed no adverse health effects. Behavioral performance correlated with the degree of water restriction, supporting the importance of controlling access to water. These behavioral paradigms can be combined with cellular resolution imaging, random access photostimulation, and whole cell recordings.

## Introduction

Neural circuits are composed of defined neuronal populations that are connected in a highly specific manner. A central goal of modern neuroscience is to link the dynamics of these neural circuits to behavior [1]. Deciphering the logic of neural circuits thus requires cell-type specific neurophysiology and manipulation [2]. Because of the wide availability of transgenic mice that allow cell-type specific targeting, the mouse is a leading model system for mammalian circuit neuroscience [3].

Over the last fifty years, experiments in behaving primates have led the way in separating causation from correlation in neurophysiological experiments. Head-fixation and body restraint have been critical because they facilitate stimulus control and measurement of movement. Non-human primates can be trained in sophisticated tasks that isolate specific brain functions. Repeated trials, often many hundreds per day, unleash powerful statistical methods to relate behavior and neurophysiological measurements. Although head-fixed monkeys have been the ‘gold standard’ system in relating the dynamics of individual neurons to behavior, cell-type-specific measurements [4], [5] and manipulation remain exceptional in non-human primates.

In contrast, in the mouse brain, cell-type-specific neurobiology is becoming routine. Transgenes can be targeted to specific types of neurons, which are nodes of the circuit diagram [2]. These transgenes can be used to identify cell-types during recordings and to manipulate circuit nodes during behavior. Mice also have a rich behavioral repertoire involving many basic sensory, cognitive and motor functions. Mice are relatively cheap, promising high-throughput approaches to neurophysiology. The microcircuit organization of the brain, as far as it is known, is similar in mice and other higher mammals. Finally, the lissencephalic macrostructure of the mouse brain allows unobstructed access to a large fraction of the brain for neurophysiology and imaging [6], [7].

Over the last decade, inspired by experiments on behaving primates, increasingly sophisticated procedures for quantitative head-fixed behaviors have been developed for mice (for a review of the literature on head-fixed behaving rats see [8]). For example, learning in the vestibulo-ocular reflex, long studied in monkeys, has been successfully probed in mice [9]. Head-fixation is critical because precise control of head motion with respect to visual stimuli is essential, as is measurement of eye position. Beyond reflexive behavior, mice have also been trained in choice-based tasks using operant conditioning. Head-fixed mice have been trained to discriminate odors [10], [11], auditory stimuli [12], visual stimuli [13]–[16], and tactile cues [7], [17]–[25]. Head-fixed mice can navigate simple mazes in a visual virtual reality environment [26]. As in most primate studies, in these types of experiment mice are motivated by thirst.

In this paper, we describe procedures for water restriction and behavioral training. We illustrate the procedures with detailed training protocols for head-fixed mice performing whisker-based tactile behaviors. Rodents use their whiskers to detect and locate objects when moving through an environment [27], [28]. The measurement of the locations of object features is a critical aspect of object identification and navigation. Inspired by previous work in freely moving rats [29], we have trained head-fixed mice to locate an object (a vertical pole) near their heads with their whiskers [7], [17]–[23]. This is by construction an active sensation behavior: mice have to move their whiskers in an intelligent manner to collect information about the world. High-speed imaging of whisker position, facilitated by head-fixation, reveals the whisker movements underlying discrimination [30]. Changes in whisker shape, caused by contact between whisker and object, report the mechanical inputs to the somatosensory system. The object-localization task is ideally suited to probing the neural basis of tactile spatial perception and sensorimotor integration [31].

## Procedures and Results

We describe our current best practice for head-fixation, water restriction and behavioral training for head-fixed mice performing tactile behaviors. The procedures are introduced in roughly the order in which they are performed in the laboratory. We first outline the surgery and apparatus for head-fixation. We then introduce water restriction, which is critical to motivate the mice for behavioral experiments [32]. Mice are then briefly acclimatized to handling by the experimenter and to head-fixation, followed by operant conditioning. The apparatus [7], [10], [17], [23] and software (http://brodylab.princeton.edu/bcontrol) for behavior, whisker tracking (https://openwiki.janelia.org/wiki/display/MyersLab/Whisker+Tracking) [30], electrophysiology (ephus.org) [7], [18], [22], and imaging (https://openwiki.janelia.org/wiki/display/shareddesigns/Shared+Two-photon+Microscope+Designs) (scanimage.org) [19]–[21] have been described elsewhere.

### 1. Surgery and head-fixation

#### Head bar surgery

All procedures were in accordance with protocols approved by the Janelia Farm Institutional Animal Care and Use Committee. All surgeries used standard aseptic procedures. Mice (∼2–6 months old, typically males) were deeply anesthetized with 2% isoflurane (by volume in O2; SurgiVet; Smiths Medical) and mounted in a stereotaxic apparatus (Kopf Instruments). Mice were kept on a thermal blanket (Harvard Apparatus) and their eyes were covered with a thin layer of petroleum jelly. During the surgery, the anesthesia levels were adjusted to 1–1.5% to achieve ∼1/second breathing rate in mice. The scalp was cleaned with 70% ethanol and betadine. Marcaine (50 µl 0.5% solution) was injected under the scalp for topical anesthesia. Ketofen (non-steroidal anti-inflammatory drug, 5 mg/kg) was injected subcutaneously and buprenorphine (opiod analgesic, 0.05 mg/kg) was injected into the intraperitoneal cavity. A flap of skin, approximately 1 cm^2^, was removed from the dorsal skull with a single cut. The remaining gelatinous periostium was removed with small scissors. The skull was cleaned and dried with sterile cotton swabs. The bone was scraped with a scalpel or slowly turning dental drill for better bonding with the glue. The exposed skull was covered with a thin layer of cyanoacrylic glue. The head bar was positioned directly onto the wet glue. Dental acrylic (Jet Repair Acrylic) was added to cover the glue and cement the head bar in position. The head bar links the skull rigidly to the behavioral apparatus.

**Figure 1:**
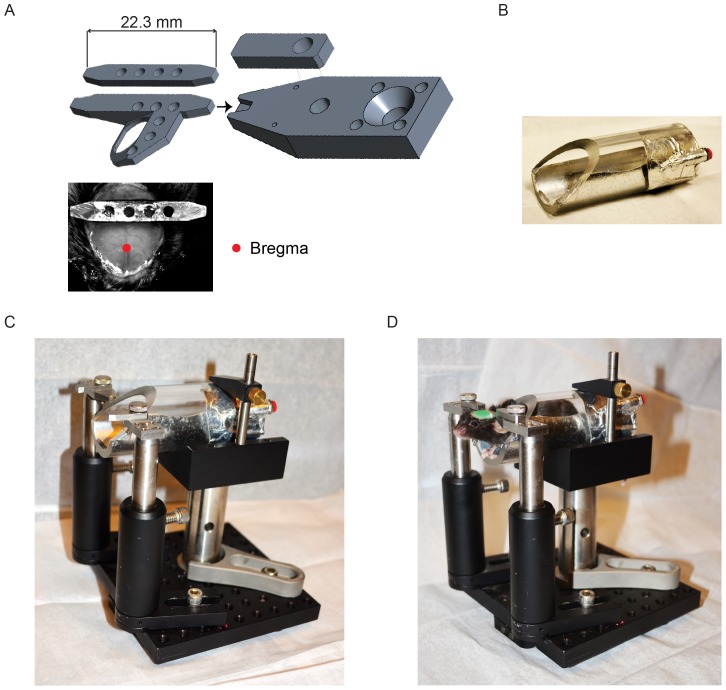
Apparatus for head-fixation. A. Left, two types of titanium head plates. Right, stainless steel head bar holder and clamp (only one of two sides is shown). The head plate is inserted into notches in the holder and fastened with the clamp (right, top) and a thumbscrew (not shown). The simple head bar (left, top) is used when access to large parts of the brain is necessary. The larger head plate (left, middle) provides better stability. The simple head bar was cemented to the skull of the mouse (left, bottom). The head of the mouse (top view) was pointing downward. The skull was outfitted with a clear skull cap [7]. The head bar was aligned at the lambda sutures. The red dot indicates the location of bregma. B. Plexiglass body tube used for head-fixed mice. Mice rest their front paws on the front ledge. The bottom of the tube is coated with aluminum foil to produce electrical contact for electric lickports. The aluminum foil is connected to the red banana socket which will be connected to electric lickports for detecting licking events. C. Example caddy used in training apparatus, assembled from standard optomechanical components (Thorlabs). The head bar holder is mounted towards the left. D. A head-fixed mouse in the caddy.

For experiments requiring maximal mechanical stability, we typically use an extended head bar, with a plate that is fitted in three dimensions to the shape of the dorsal mouse skull (**Figure 1A**). When cemented to the skull this plate bonds with all skull plates over large surface areas and thereby links the skull plates and rigidifies the skull. With the head-plate clamped to the head-plate holder, all remaining brain motion is caused by movement of the brain within the skull (data not shown). For experiments requiring access to large areas of the brain we use a minimal head bar (22.3×3.2 mm) [7].

#### Optional viral gene transfer

In some cases viral reagents, typically adeno-associated virus (AAV) were introduced during the head bar surgery [18]–[21]. Using a dental drill with an FG 1/4 drill bit, a small hole was drilled into the skull. The virus was introduced using a fine glass injection pipette (tip diameter approximately 15–20 µm) beveled to a sharp tip (outer diameter, 20–30 µm). Beveling is critical since it allows the pipette to penetrate the dura without dimpling the cortex, greatly reducing tissue damage. The pipette containing virus was lowered into the brain region of interest. Viral suspension is injected slowly into the parenchyma (10 nL per minute). Approximately 30 nL of AAV (approximately 10^12^ titer) is sufficient to transduce neurons in a 500 µm diameter column of the neocortex [33]. Following the surgery, buprenorphine (0.1 mg/kg) was administered once. Ketoprofen (5 mg/kg) was administered once a day for two days as an analgesic to reduce inflammation. Animals were examined once a day for three days for signs of infection, lethargy, and grooming.

In other cases it may be necessary to introduce viruses during training. As viral transduction efficiency can be low in water restricted mice, water should be supplemented for 2 days prior surgery (3–4 ml water per day) [26].

#### Head-fixation and lickport

For head-fixation, the wings of the head bar are seated into notches in a stainless steel holder and fixed with a pair of clamps and thumbscrews (**Figure 1A**). The mouse body is inserted into an acrylic ‘body tube’ (1⅛ inch i.d.; McMaster; P/N 8486K433) (**Figure 1B**), with the mouse head extending out and the front paws gripping the tube edge or a ledge after head-fixation. The holder and body tube in turn are attached to a caddy (**Figure 1C**). Typically, the head bar is about 30 mm above the bottom of the body tube. The caddy is fixed to the behavior box using magnetic kinematic bases (e.g. Thorlabs, KB3X3). These mounts allow the experimenter to conveniently head-fix mice outside of the apparatus in the caddy. The caddy with mouse can then be placed into the apparatus rapidly and consistently. A head-fixed mouse should crouch in a natural position in the body tube, with its paws resting on a tube edge or a ledge (**Figure 1D**).

Water rewards are provided by different types of custom-made lickports that sense the movement of the tongue. Electrical lickports are activated by the tongue making contact with the steel nozzle of the lickport [34]. Optical lickports are activated by interruptions in the light path between an LED and a phototransistor [23]. Optical lickports require regular cleaning to ensure that the optical path remains unobstructed. Electrical lickports are more robust, but can introduce artifacts in electrophysiological measurements.

The lickport position relative to the mouse is a critical parameter during training. If the lickport is too close to the mouth, the mouse might lick compulsively. If the lickport is too far, the mouse might miss rewards and become discouraged. We typically start with the lickport 0.5 mm below the lower lip, and 5 mm posterior to the tip of the nose. During training the lickport typically is moved away from the mouth to discourage compulsive licking (see **Discussion**).

### 2. Water restriction

How can we motivate experimental subjects to cooperate in behavioral experiments? In the case of human subjects, this is typically achieved by the subjects' willingness to participate in scientific experiments, or by providing subjects with economic rewards. For non-human subjects, experimenters can restrict the animal subjects' access to basic needs such as food and water [35]–[39], and use them as rewards during behavioral experiments. Rodents generally cope better with water restriction than food restriction [40]. In an attempt to use food restriction (2–3 grams of solid food per day with free access to water) some mice developed significant health problems (high health scores) before reaching 15% weight loss. Here we describe procedures for motivating mice by limiting their access to water, based on pioneering work by Slotnick and colleagues in the context of freely moving olfactory behavior in mice [32], [41]. Although most tested mice were male, females showed similar weight loss and behavioral performance after water restriction. On days when behavioral experiments were carried out, mice typically obtained all of their water during performance in the behavior apparatus (approximately 1 ml water per day). On other days, including weekends and holidays, mice received 1 ml water per day.

Water restriction was started after mice recovered from surgery (at least three days after surgery). Mice were housed singly in cages containing tunnels and bedding material, in a reverse light cycle room. Housing in small groups of siblings is also possible. Training and behavioral testing occurred mainly during the dark phase. Relative humidity critically affects the animals' need of water [42] and was kept at 40–50%, with little seasonal variations. Following full and complete recovery from a previous surgery (at least three days post surgery), mice were placed on a water restriction schedule in preparation for behavioral conditioning. Dry food was continuously available (Rodent diet 5053). One ml of water was dispensed manually into bowls which were attached to the inside walls of individual cages, at consistent times of day. Mice consumed this water within minutes. This corresponds to approximately 35% of ad libitum water consumption for C57BL/6J mice (Mouse Phenome Database from the Jackson Laboratory: http://www.jax.org/phenome).

**Figure 2:**
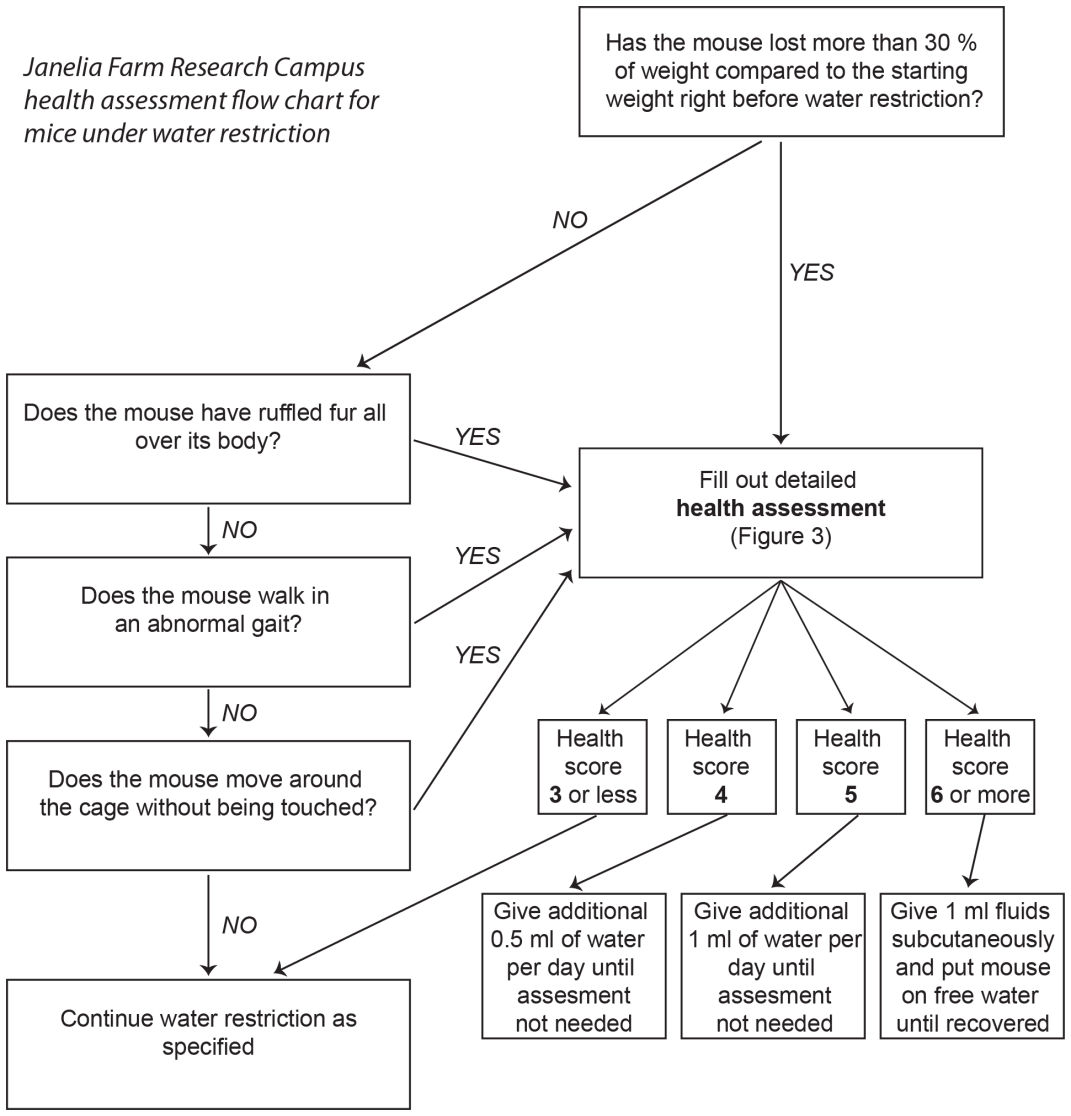
Flowchart for monitoring mice under water restriction.

**Figure 3:**
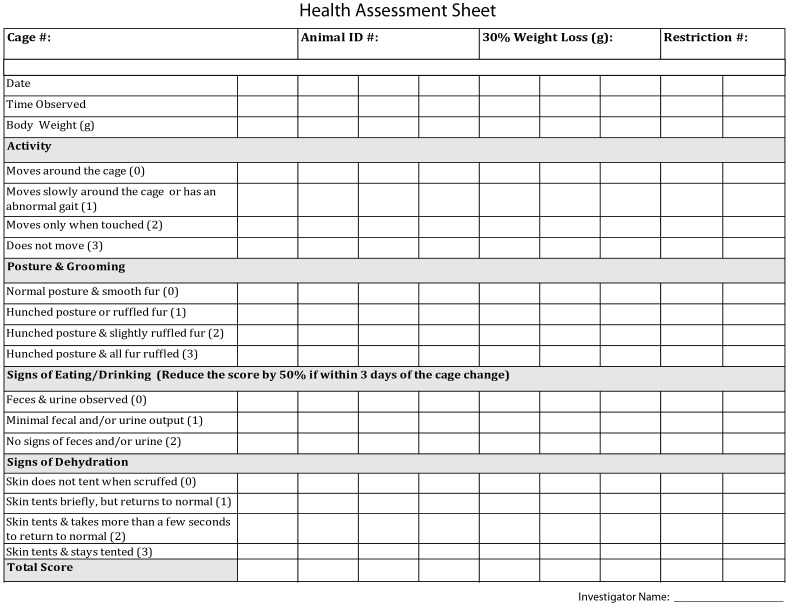
Mice with one or more indicators of stress or pain are placed on detailed health assessment. Activity levels, grooming, and indicators of eating and drinking are scored daily in a health assessment sheet. The total aggregate health score determines if mice are supplied with additional water (see flowchart in Figure 2).

**Figure 4:**
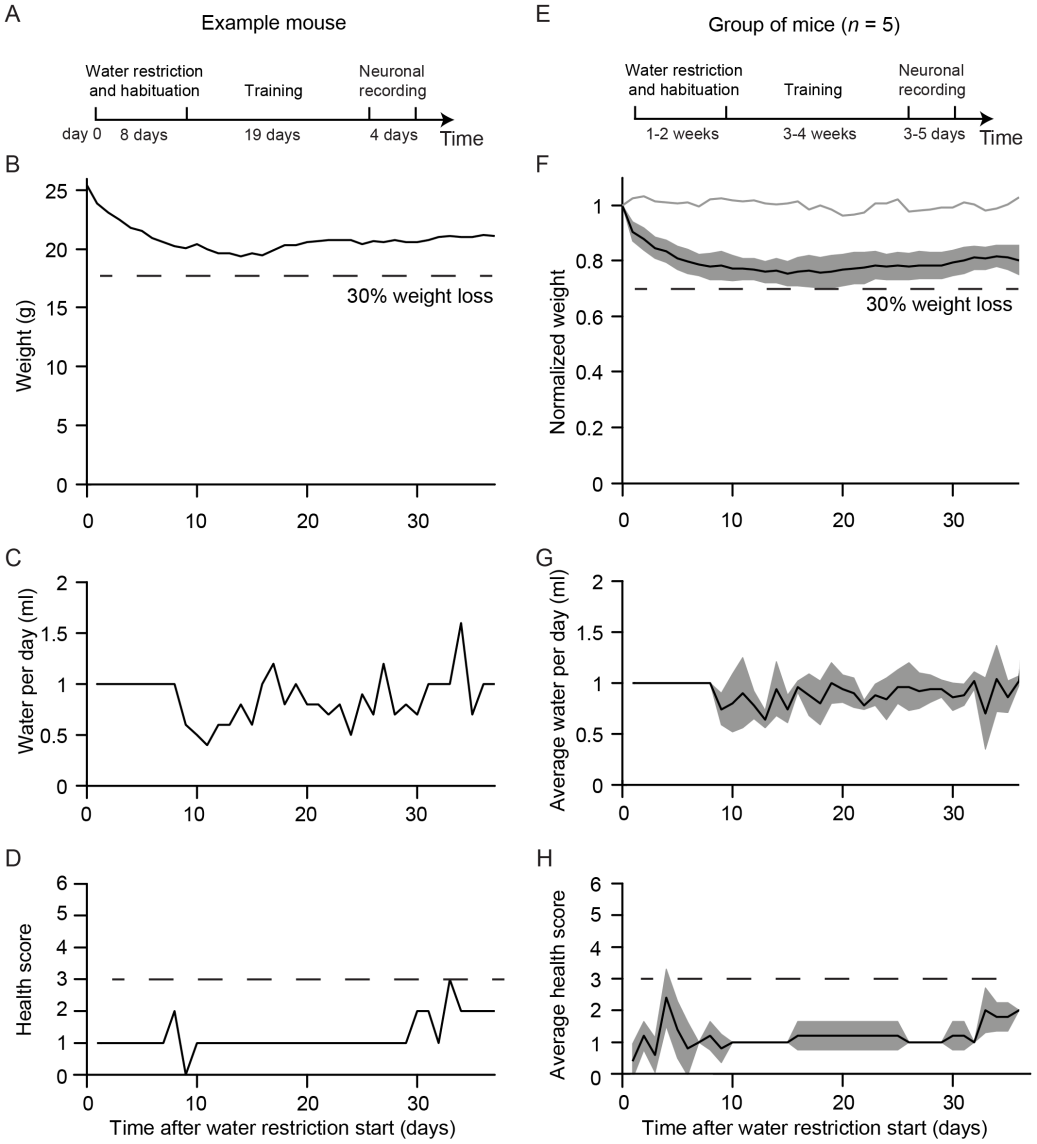
Mouse weight and health during water restriction. All mice were trained in a lick/no-lick object location discrimination task using a single whisker (same mice as in Figures 2 & 3 of [18]). Rewards consisted of approximately 8 µl of water per trial. A. Experimental time-course for one example mouse, from the beginning of water restriction to the end of electrophysiological recordings. An 85 day old mouse (25.4 g) was put on water restriction for eight days, followed by training (starting on day 9) and recording (starting on day 28). B. Body weight as a function of time. Same mouse as in A. The dashed line indicates 30% weight loss. C. Water consumed per day. After start of training mice mostly received their water during the training session. A larger number of correct trials will lead to more consumed water. Same mouse as in A. D. Health score as a function of time. A health score larger than 3 (dashed line) triggers more detailed evaluation and possibly water supplements. Same mouse as in A. E. Experimental time-course for a group of 5 mice. Same format as A. F. Average body weight of 5 mice (black line) and 2 mice with free access to water (grey line). Shading indicates standard deviation. Experimental time-course for all mice was similar, but not identical to A. G. Average water consumed. H. Average health score.

All mice undergoing water restriction were monitored daily for hydration, weight, ruffled fur, and movement (**Figure 2**). The pre-restriction body weight is typically in the range 23–30 g for 2–6 months old males. If mice drop below 70% of pre-restriction weight, or if mice show signs of dehydration or pain, their health is assessed in more detail. The health assessment is summarized in a health score (**Figure 3**). Health scores in the range of 1–2 typically reflect slightly reduced activity and ruffled fur around the margins of the head bar surgery. If the health score is above three, mice receive supplemental water (**Figure 2**). After stabilization of body weight, typically after seven to ten days of water restriction, the training procedure began (**Figure 4**). The body weight tends to increase with long periods of restriction after the initial dip (**Figure 4B,F**). With shorter periods of water restriction, mice will not be sufficiently motivated to overcome fear-related reflexes, triggered by new environments that are invariably part of initial stages of training. Without strong motivation, mice often stop working after a few trials and may learn undesired behaviors. Trained mice often receive all of their water (1 ml, sometimes more; **Figure 4G**) during performance in the behavioral apparatus. After behavioral sessions in which mice consumed little water (<0.5 ml) a water supplement (0.2–0.5 ml) was typically provided to a total water consumption of 0.6 ml per day or more.

**Figure 5:**
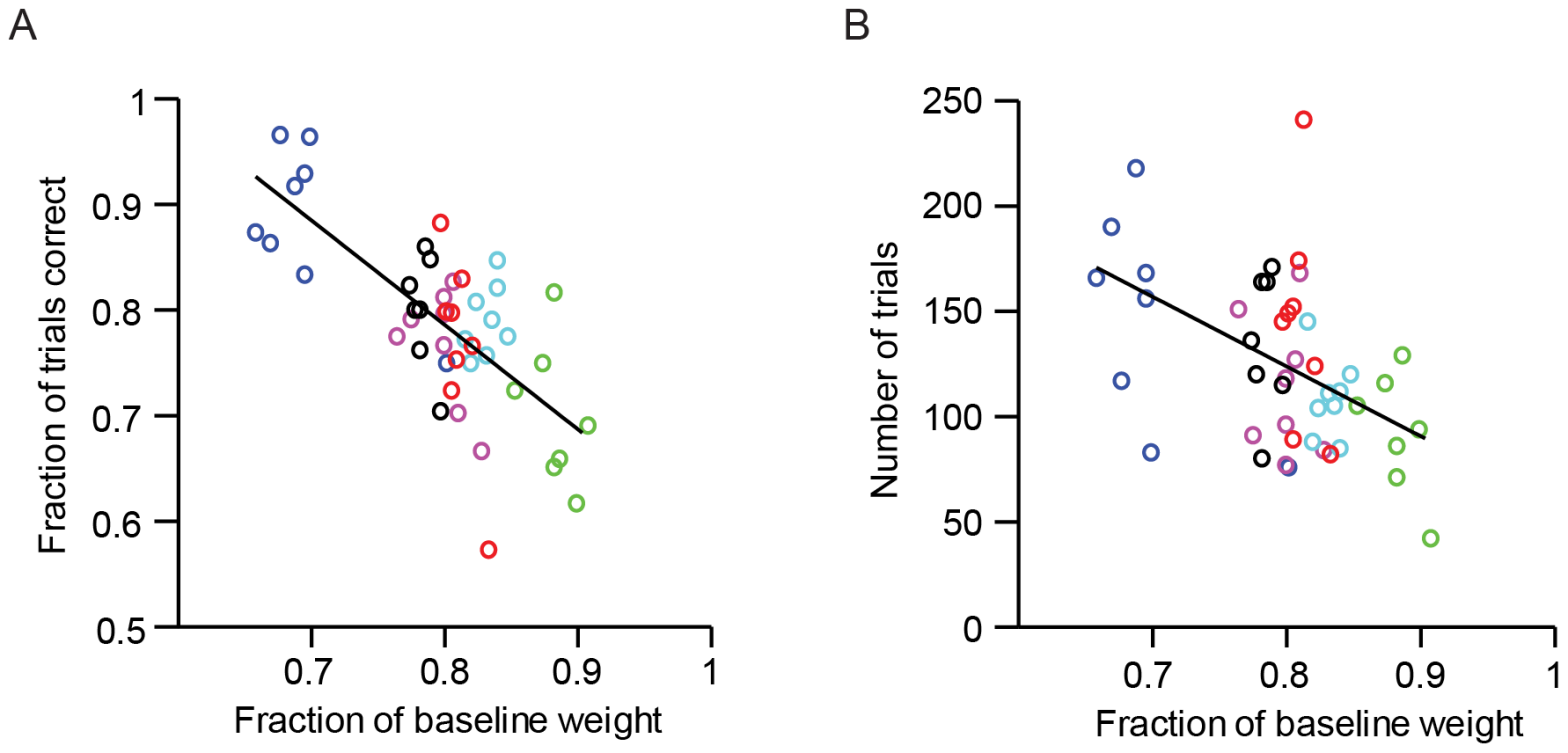
Performance as a function of normalized body weight. A. Performance as a function of normalized body weight. Each circle corresponds to one behavioral session. Different colors correspond to different mice (7–8 sessions per mouse). The sessions included are the first seven to eight sessions of discrimination training (corresponding to the training phase shown by open symbols in Figure 3a of [23]. Multiple factors can compromise performance in behavioral experiments. In this experiment mice were trained in serial with individualized attention to reduce variability due to uncontrolled factors. The correlation coefficient is R^2^ = 0.52 (*p*<0.001). B. Number of trials as a function of normalized body weight. Mice usually perform less trials in the first few sessions of training. Same sessions as in (A). The correlation coefficient is R^2^ = 0.24 (*p*<0.001).

**Figure 6:**
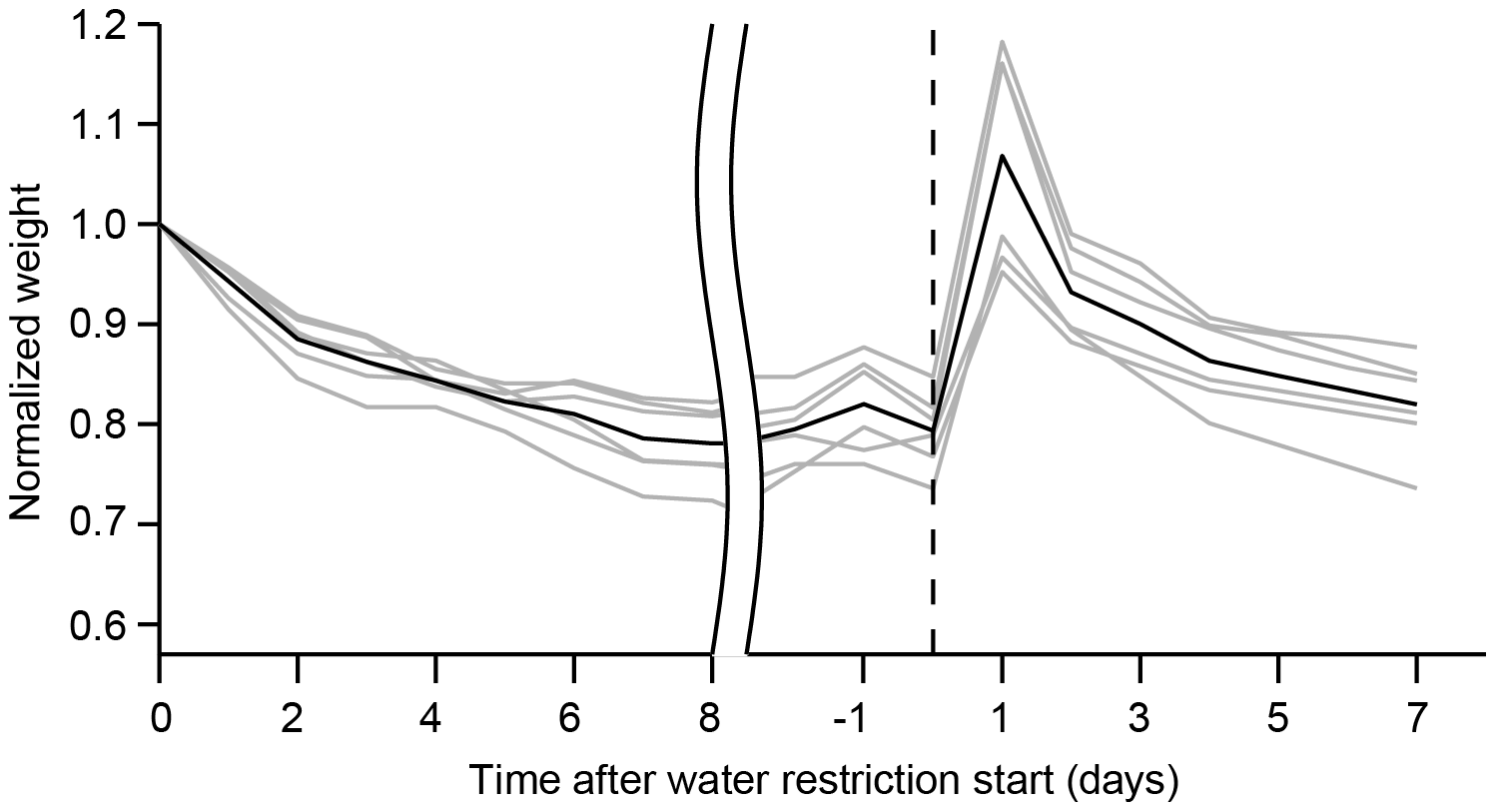
Normalized weight of 5 female mice after the initial water restriction (left) and after one day of free access to water (dotted line, day 0).

At steady state, mice typically lose 20% of body weight compared to age-matched controls (**Figure 4B, F**) while consuming 1 ml of water per day. Our experience has shown that mice must lose at least 15% of body weight to be motivated to perform challenging behavioral tasks for large numbers of trials. During early stages of training the number of trials performed per session, as well as the fraction of correct trials, correlate with weight loss (**Figure 5A, B**). This indicates that water restriction determines the mouse's motivation and drives learning and performance. Consistent water restriction, including weekends, is critical. This is because even one day of free access to water causes substantial weight gain (**Figure 6**) and loss of motivation for several days.

Under our conditions health scores remain in a normal range (<3) for four months of continuous water restriction (see **Training the lick-left/lick-right task with a delay epoch**). Higher scores are typically related to other factors, such as stressful surgeries, large head-implants, or infection. We performed a histological analysis for 6 male C57Bl/6J mice after one month of water restriction. Most organ weights, including heart, spleen, kidneys, adrenal glands, and testes, were indistinguishable from control mice (6 male mice; *ad libitum* water consumption). The brain (94±1% of control, mean ± SD, p<0.001, t-test; all tests with Bonferroni correction) and spleen (54.6±6.7%, p<0.001) were smaller in the water deprived mice. Water restricted rodents tend to have lower organ weights [43]. The reason for the pronounced reduction of spleen size is unknown.

Blood samples were further extracted to analyze the physiological state of water restricted mice. The concentrations of most solutes were in the normal range, including sodium, potassium, chloride, aspartate aminotransferase (AST), alanine aminotransferase (ALT), blood urea nitrogen (BUN), CO_2_, total protein, albumin, tibili and creatinine. Glucose (55±16%; p<0.01) and alkaline phosphatase (ALP) (67±18%; p<0.05) were reduced in the water-deprived mice. Mice eat when water is available. The reduced glucose and ALP likely reflect that the mice were euthanized long after eating.

### 3. Handling and head-fixation

Four days prior to instrumental training (at least three days after starting water restriction) mice should be handled so that they become habituated to the training environment, including the experimenter's hands, body tube, head-fixation, rig, sounds in the experimental room, and other factors. As a result mice will be less stressed and learn faster. Here we describe our current procedures, but procedures with less extensive habituation have also been successful [23].

Handling proceeds in three steps, typically on successive days.

Day 1. The mouse is acclimatized to the experimenter's hands. We typically start by placing two sunflower seeds into the mouse's holding cage for 10–15 minutes, while removing any objects that the mouse can hide in (tubes, running wheel, cotton nests, etc). After the agitated mouse has settled down, we corner it with our hands with deliberate and gentle movements and allow the mouse to climb on our hand. We hold the mouse in our hands for 5–10 minutes until it calms down, as evidenced by grooming behaviors, and offer the mouse water using a syringe (approximately 0.2 ml). Drinking is a sign of relaxation.

We then let the mouse explore the body tube until he enters it. If the mouse enters the body tube we repeat the procedure 4–5 times without forcing the mouse. Otherwise we try again on Day 2.

**Figure 7:**
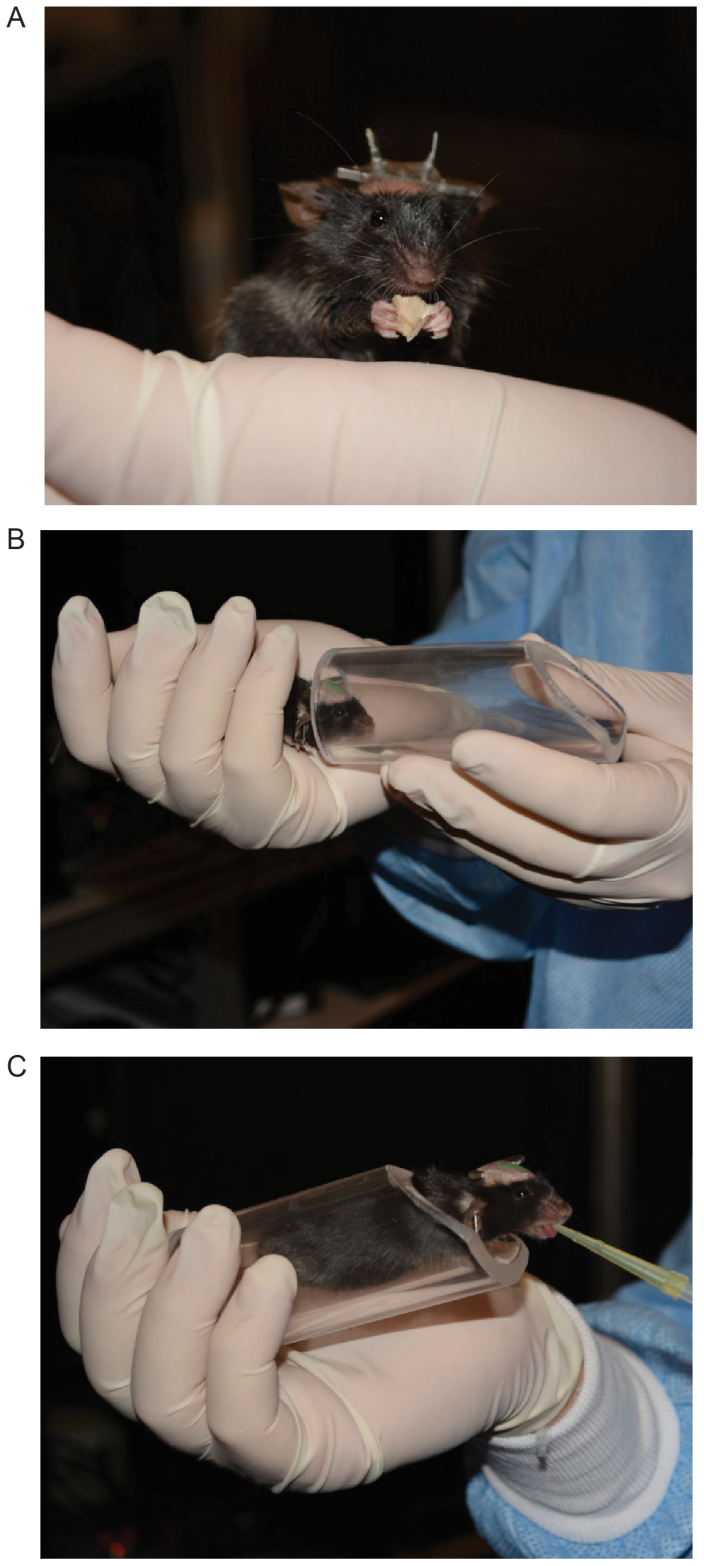
Key stages in mouse handling. A. Mouse eating a sunflower seed on the experimenter's hand. The pins emanating from the top of the mouse head correspond to ground and reference electrodes for extracellular recordings. B. Mouse being familiarized with the body tube. C. Mouse receiving a water reward in the body tube.

Day 2. The mouse is further acclimatized to the experimenter's hands and the apparatus. We hold the mouse and have it nibble at a sunflower seed (**Figure 7A**). The mouse will eat only if he feels comfortable. The mouse then explores the body tube again. A water reward (0.1–0.2 ml) is given after the mouse has entered the tube (**Figure 7B, C**). At this point the mouse is head-fixed rapidly (<10 s), with its body in the holding tube. Additional water (0.2 ml/5 minutes) is provided during head-fixation (10–15 minutes).

Day 3. The mouse is acclimatized to the apparatus. The mouse is head-fixed and the caddy is placed into the behavioral apparatus for 30 minutes. Water rewards (0.2 ml) are provided every few minutes, for a total of 1 ml.

Day 4. The procedures from the third day are repeated, but extended to 45 minutes. In addition, the mouse is introduced to a lickport as a source of water.

### 4. Training the lick/no-lick object location discrimination task

In this section we describe training of one version of a lick/no-lick (go/no-go) object location discrimination task in the dark (corresponding to the data in **Figures 4** **,** **8** **,** **9**). The goal is to train mice to use a single whisker (typically C2) to locate a vertical pole for a water reward. Single whisker tasks greatly simplify linking sensory stimuli to behavior and neurophysiology [18].

**Figure 8:**
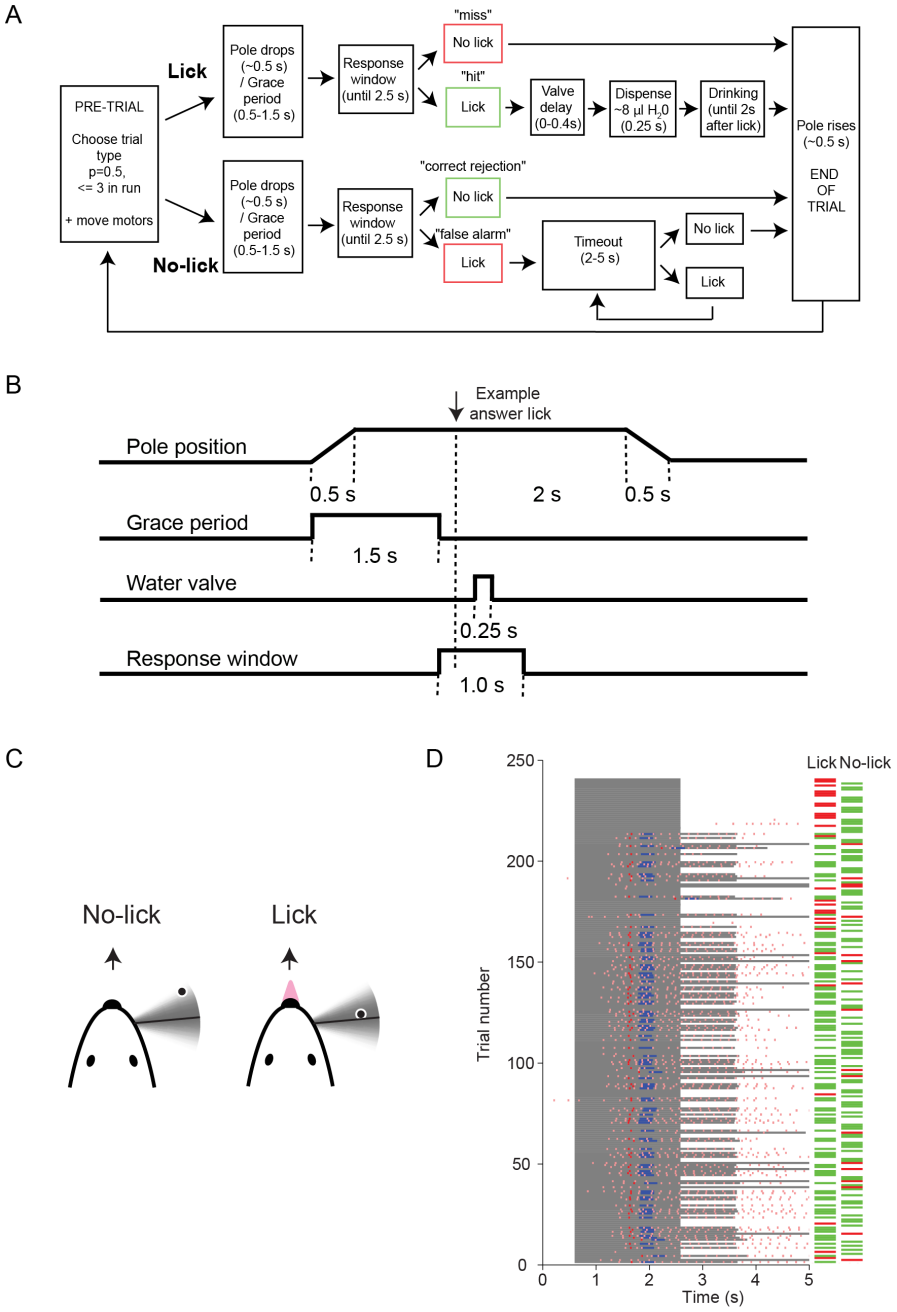
A lick/no-lick object location discrimination task for head-fixed mice [23]. A. Block-diagram of the possible events in a single trial. B. Schematic representation of event timing during a single lick trial. C. Schematic representation of the behavioral contingency. Mice had to lick for a water reward when the pole was in a posterior position and hold their tongue when the pole was in an anterior position. In some experiments, the contingency of the pole positions was reversed. D. Behavioral data from one session. The abscissa shows the time from trial start. Lick and no-lick trials are randomly interleaved. The pink ticks indicate licks. The red ticks indicate the first licks after the grace period. The blue bars correspond to the open times of the reward water valve. The horizontal green and red bars indicate whether each trial is correct or incorrect, respectively. The dark gray shading indicates that the pole is fully descended and in reach of the whiskers.

**Figure 9:**
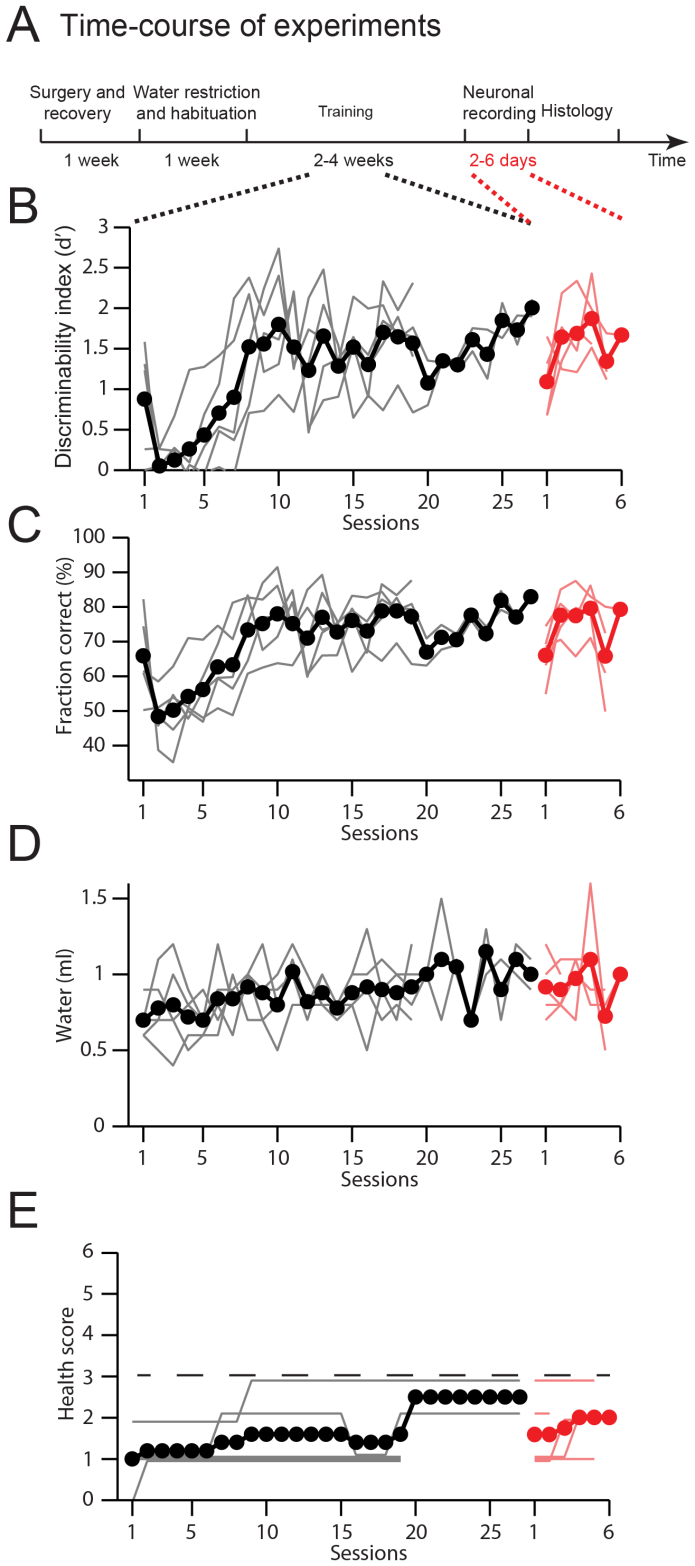
Performance of the lick/no-lick object location discrimination task. A. Time-course of experiments. B. Learning curves showing the discriminability index, d'. Thin lines correspond to individual mice. Thick lines, average. Red, recording sessions. C. Learning curves showing the fraction of correct trials. D. Water consumed. E. Health score. A health score larger than 3 (dashed line) triggers more detailed evaluation and possibly water supplements.

During each trial the object, a vertical pole (0.5–1 mm in diameter), was presented at one of several possible positions on one side of the face (**Figure 8C, D**). The no-lick position was a single anterior pole location. The lick position was one, or optionally multiple [19], [20], relatively posterior pole locations. In some experiments the contingency was reversed. The distance of the posterior pole location to the whisker pad was 5–8 mm. The final distance between the no-lick and the most anterior lick position was 4.29 mm along the anterior-posterior axis. Water was available through a single lickport centered on the midline. Movement of the pole took 0.5 s, after which the animal was given 2.5 s to search for the object with its whisker and indicate object location by licking or withholding licking (**Figure 8A, B**). To encourage multiple whisker-object contacts before signaling a response, the animal was given a grace period (0.5–1.5 s) from onset of pole movement where licking did not signal the response outcome. Following the grace period, a lick in the remaining pole availability time (answer lick) was scored as a hit if the pole was in a lick position or a false alarm if the pole was in the no-lick position. Hits triggered opening of a water valve to deliver approximately 8 uL of water. Two seconds after the answer lick, the pole retracted and the intertrial period began. On false alarm trials the mouse was given a timeout, typically 2–5 s, which retriggered on any additional licks during the timeout. If no lick occurred during the response window, the trial was scored as a miss (lick trial) or a correct rejection (no-lick trial). On both misses and correct rejections the intertrial period began immediately following the end of the response window. The intertrial period typically lasted two seconds, during which the pole first moved to the midpoint of the two pole positions and then to the position of the next trial.

Training proceeded through multiple stages. Mice were trained once a day for sessions lasting 45 to 90 minutes. The first day of training began with association between the presence of the pole and water availability. The pole was moved into the center of the whisker field (to ensure whisker-pole contacts) and any licking triggered a water reward. After three lick-triggered rewards the protocol was paused and the pole was moved out of reach of the whiskers. After a 10 s delay, the process was repeated, until mice licked concurrently with touch between whiskers and pole. If the mouse failed to lick after one minute, the lickport was manually seeded with a water droplet by briefly opening the valve using the behavioral control software. Mice often lick when smelling the water emerging from the lickport. If the mouse still refused to lick, the lickport was moved closer such that the droplet touched the fur. This always caused the animal to lick.

Mice were then exposed to the timing of the trials. The pole was moved to a single ‘lick’ position on repeated trials. Mice received rewards when licking 1–2 s after the pole came within reach and were not punished for excessive licking. Once the mouse received rewards on five consecutive trials, the pole was introduced in the no-lick position on 20% of trials. The initial no-lick position was far anterior, out of reach of the whiskers. This specifically links detection of the pole within the whisker field, rather than other cues such as sound and vibration, to availability of reward. Once the mouse licked on >75% of lick trials the probability of the no-lick position was increased to 50%, with a maximum of three consecutive trials of a single type. In cases of five or more consecutive misses, the no-lick probability was reduced to 0% until the animal began responding. About one half of the mice progressed to the 50% no-lick probability stage by the end of the first day of training, whereas others had difficulty moving beyond the initial association of pole presence and water availability.

Prior to the second day training session all whiskers except C2 were trimmed to 3 mm in length (i.e. too short to contact the pole). The lick (go) location was positioned 2 mm anterior to the resting position of the C2 whisker for each mouse, whereas the no-lick (no-go) position was out of reach. The pole was placed randomly in lick and no-lick positions with 50% probability, with a maximum of 3 consecutive trials of a single type. Whisking and licking were examined to identify possible training failure modes for each mouse. In case of high miss rates on trials where the whisker touched the pole, the lickport position was adjusted to ensure it was triggered properly on each attempted lick. If the animal had a high miss rate and the whisker did not strike the pole, the pole location was moved closer to the resting position of the whisker. If the animal was licking compulsively on lick and no-lick trials, the lickport was moved further from the animal's mouth and/or the no-lick probability was increased to 80% until several correct rejection trials occurred. If the animal was licking cautiously at least once on both trial types to probe for water rewards the timeout punishment was increased to 5 s. As the performance of the mouse increased during or across sessions, the no-lick position was progressively moved toward the lick position, within easy reach of a vigorous whisk of the C2 whisker, making this an object location discrimination task. The final distance between the lick position and the no-lick position was 4.29 mm. Sessions were terminated when mice missed 10 lick trials in a row (even after adjusting the lickport position for the early training sessions).

**Figure 10:**
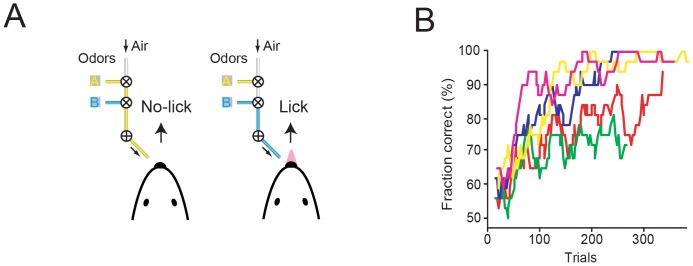
A lick/no-lick olfactory discrimination task for head-fixed mice. A. Schematic representation of the behavioral contingency. Mice had to lick for a water reward when odor B was presented and hold their tongue when odor A was presented. B. Performance in the first session of the odor discrimination task (data from [10]). Colored lines correspond to individual mice (*n* = 5).

Individual mice learn at a variety of rates. After one week of training, the best mice achieved peak performance of >90/100 consecutive trials correct, with total session performance of >80% correct (discriminability index, d' >2), whereas other mice required up to 3 weeks to achieve similar performance levels (**Figure 9A–C**). In our experience, object localization with single whiskers is challenging for mice, and the training time might reflect the inherent difficulty of the task. With one row of intact whiskers training times are much shorter: mice typically learn the lick/no-lick pole detection task in 1–3 days [21]. Even faster learning can be achieved in lick/no-lick olfactory discrimination behaviors. We have found that mice routinely learn to report two different odors within one session [10] (**Figure 10**).

We have also observed that the distance of the pole from the whisker pad has a large impact on performance. The whisker is linearly tapered and its bending stiffness decreases gradually with distance from the whisker pad over five orders of magnitude [17], [44]. Forces exerted by the pole on the whisker are usually larger when the pole is closer to the whisker pad, leading to faster learning in mice. In our experiments the distance of the pole to the whisker pad was 5–8 mm. Future innovations in shaping mouse behavior will no doubt shorten training times.

On days with behavioral sessions, mice generally obtained all water for the day during the session and were allowed to perform until sated. Mice typically performed 300 trials and received 0.6–1.2 mL of water. The amount of water consumed was determined by weighing the mouse before and after the session (including any excrement). If the mouse consumed an unusally small volume of water (<0.5 ml) a small water supplement (0.2–0.5 ml) was provided a few hours after training. Mice maintained body weight with health scores in the normal range (<3; **Figure 9D, E**).

### 5. Training the lick-left/lick-right task with a delay epoch

The lick/no-lick object location discrimination task described above has several disadvantages for the study of decision making. First, animals are biased towards licking. Second, sensation and action (i.e. the answer lick) happen nearly simultaneously. For numerous experiments it is of interest to separate “sensation” and “action” in time. We therefore designed a task in which both pole positions are rewarded, with a delay epoch that separates sensation and action. The temporal structure of the task was modeled after behavioral paradigms widely used in psychophysics [45].

**Figure 11:**
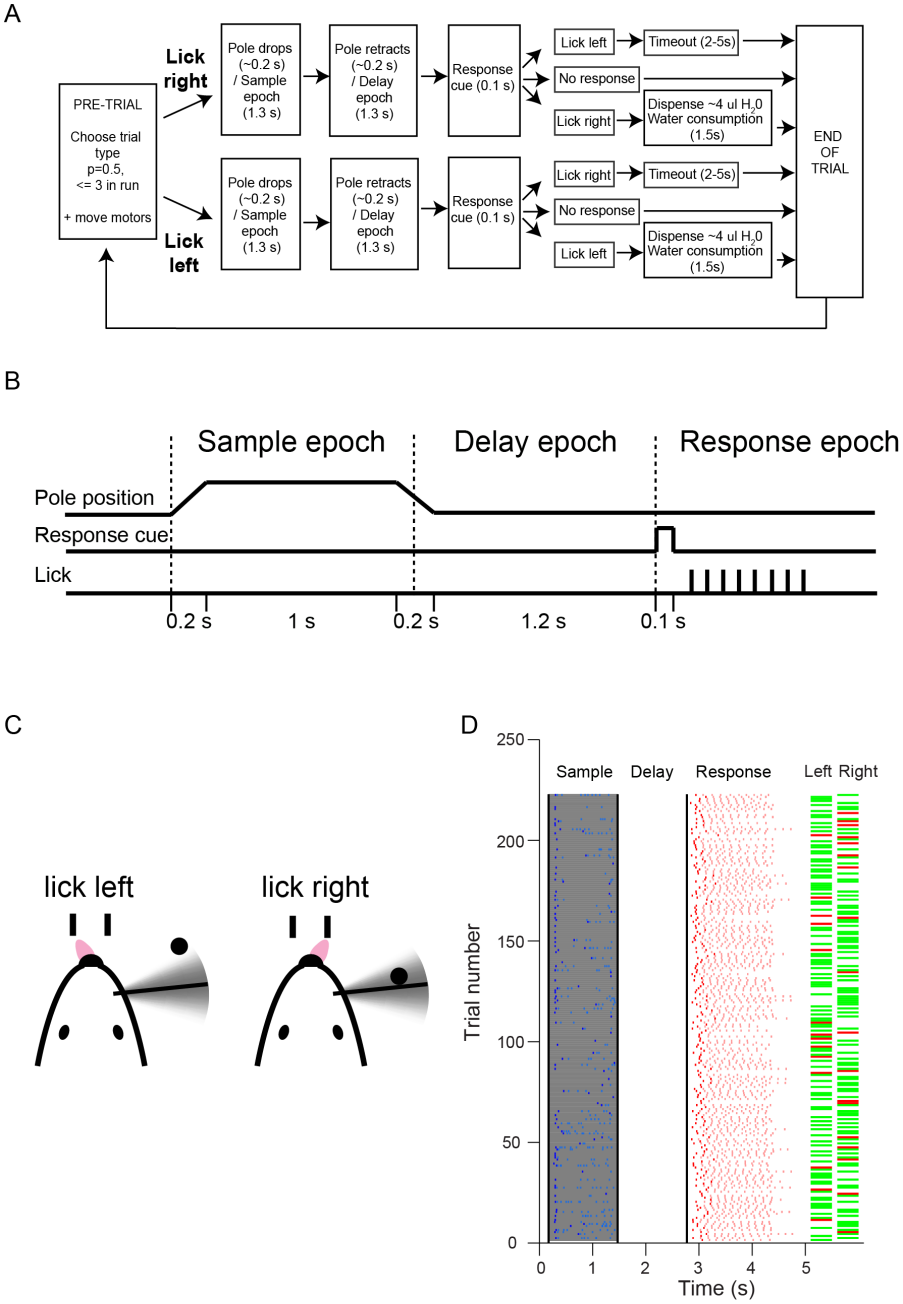
A lick-left/lick-right object location discrimination task with a delay epoch [7]. A. Block-diagram showing the possible events in a single trial. Licking during the sample or delay epochs leads to a brief timeout (1–1.2 s) and were not shown for clarity. B. Schematic of event timing during a single trial. Same as Figure 1C of [7]. C. Schematic representation of the behavioral contingency. Mice had to touch a left lickport for a water reward for an anterior pole location and a right lickport for a posterior pole location. In some experiments the contingency of the pole positions was reversed. D. Behavioral data from one session. Trials with the licking response before the response cue were excluded for clarity (25% of total trials). The abscissa shows the time from trial start. Lick-left and lick-right trials are randomly interleaved. The blue and light blue ticks indicate the onset time of the first and subsequent contacts respectively. The red and pink ticks indicate the first and subsequent licks respectively. The horizontal green and red bars indicate whether each trial is correct or incorrect respectively. The dark gray shading indicates the sample epoch during which the pole is within reach of the whiskers. The black vertical lines delineate the sample, delay and response epochs.

Mice were trained to perform a symmetric response lick-left/lick-right object location discrimination task with a short-term memory component (**Figure 11**) [7]. The behavioral apparatus and training procedures have been described [7]. In short, mice need to use their whiskers to locate a vertical pole (0.9 mm in diameter), presented at one of two possible positions on the right side of the face. The posterior pole position was placed 5 mm from the whisker pad. The two pole positions were spaced 4.29 mm apart along the anterior-posterior axis (40 degrees of whisking angle) and were held constant from session to session. Water was available through two lickports, spaced 4.5 mm apart. Mice were trained to indicate the posterior pole position with licking right, and the anterior pole position with licking left (**Figure 11C**); in some experiments the contingency was reversed. The pole was only available to the animals during the sample epoch and the animals need to hold their response for a brief delay epoch (**Figure 11B**). The delay epoch thus separated “sensation” and “action” in time. At the beginning of each trial, the vertical pole quickly moved within reach of the C2 whisker (0.2 s travel time). The pole remained within reach for 1 s, after which it was retracted. The retraction time was 0.2 s, of which the pole remained within reach in the first 0.1 s. The delay epoch lasted for another 1.2 s after the completion of pole retraction (delay epoch, 1.3 s total, **Figure 11B**). At the end of the delay epoch, an auditory “response” cue (pure tone, 3.4 kHz, 0.1 s) was issued.

**Figure 12:**
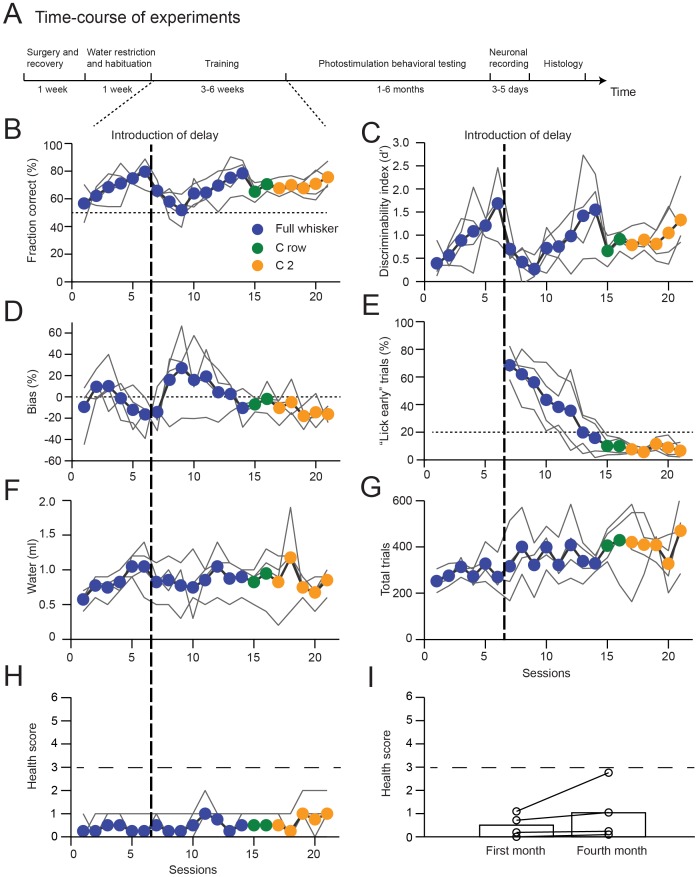
Performance of the lick-left/lick-right object location discrimination task with a delay epoch (data from Figure S1 [7]). A. Schematic of time-course of experiments. B. Learning curves showing the performance. Thin lines correspond to individual mice. Thick lines, average. Colors correspond to whisker trimming. Vertical dashed line indicates when the delay epoch was introduced. The four mice were from the same litter (2 males and 2 females). Same as Figure S1B in [7]. C. Learning curves showing the discriminability index, d'. D. Bias: performance of lick-right trials minus performance of lick-left trials. Same as Figure S1C [7]. E. The fraction of trials with licking responses during the sample or delay epoch. Same as Figure S1D [7]. F. Water consumed. G. Trials per session. H. Health score. A health score larger than 3 (dashed line) triggers more detailed evaluation and possibly water supplements. I. Health score for four mice that were under water restriction for four months. A health score larger than 3 (dashed line) triggers more detailed evaluation and possibly water supplements.

Training was carried out in daily behavioral sessions that lasted 1–1.5 hours [7]. In the first behavioral session, mice received liquid rewards simply by licking either lickport. The auditory “response” cue was played immediately before water delivery; this contingency was kept constant throughout training. In the following sessions, the vertical pole was presented to indicate the rewarded lickport (e.g. the pole presented to the posterior position indicated that the right-side lickport was rewarded, see **Figure 11C**). The rewarded lickport alternated between the two lickports after three rewards. Occasionally, water delivery by manually clicking a computer -controlled valve was necessary to prompt the mice to lick the other lickport. This phase of training lasted for 1–3 sessions. Presentation of the pole allowed the mice to gradually associate a pole position with licking the correct lickport. Presentation of the pole at the posterior position always touched some of the whiskers, whereas presentation of the pole at the anterior position made fewer contacts. Often, mice would start to associate the pole with licking the correct lickport. Signs of this could be gauged by the observation that mice quickly switched to lick the right-side lickport when the pole was presented at the posterior position (which typically contacted their whiskers). Once such signs were observed, mice were subjected to the object location discrimination task with no delay epoch, in which the presentation of the pole position was randomized. The mice were free to lick the correct lickport immediately after the pole was presented. Licking before the “response” cue was not punished. Licking the incorrect lickport after the “response” cue led to no liquid reward and a brief timeout (2–5 s). Typical mice learned this step quickly (5 sessions, **Figure 12**). After mice reached criterion performance with full whisker fields (typically >75% correct), the delay epoch was introduced. First, mice were trained to lick only after the “response” cue. Licking before the “response cue” was punished by a loud “alarm” sound (siren buzzer, 0.05 s duration, 2–4.5 KHz, 102 dB without shielding, RadioShack, 273-079), followed by a brief timeout (1–1.2 s). Continued licking triggered additional timeouts. The trial was allowed to resume once the timeout was complete, but these trials were excluded from the analyses (“lick early” trials, **Figure 12E**). Mice gradually learned to suppress their licking before the “response” cue. Once mice were successfully conditioned to lick following the “response” cue, the pole was removed at the end of the sample epoch and the delay epoch was added in incremental steps (typical steps of 0.2–0.4 s added once per session).

After mice achieved criterion performance (>70%) on the object location discrimination task with a delay epoch, their whiskers were progressively trimmed (full whiskers→C row→C2, see **Figure 12**). The total training time for the full task is 3–4 weeks (**Figure 12A–D**). Trials in which mice did not lick within a 1.5 second window after the “response” cue were counted as “ignore” and excluded from the analyses. These “ignore” trials were rare and typically occurred at the end of a session, signaling that the mouse was sated or tired. Sessions were terminated when signs of fatigue were observed (e.g. reduced whisking, occurrence of “ignore” trials). Typically, the last 20 trials within each session were excluded from analyses. In a typical experimental session, fully trained mice performed 400 behavioral trials (**Figure 12G**). Under our conditions animals typically receive 0.8–1 ml water per day during training (**Figure 12F**). The health scores remain in a normal range (0–3) for up to four months of continuous water restriction (**Figure 12H, I**).

### 6. Modifications of the lick-left/lick-right task

**Figure 13:**
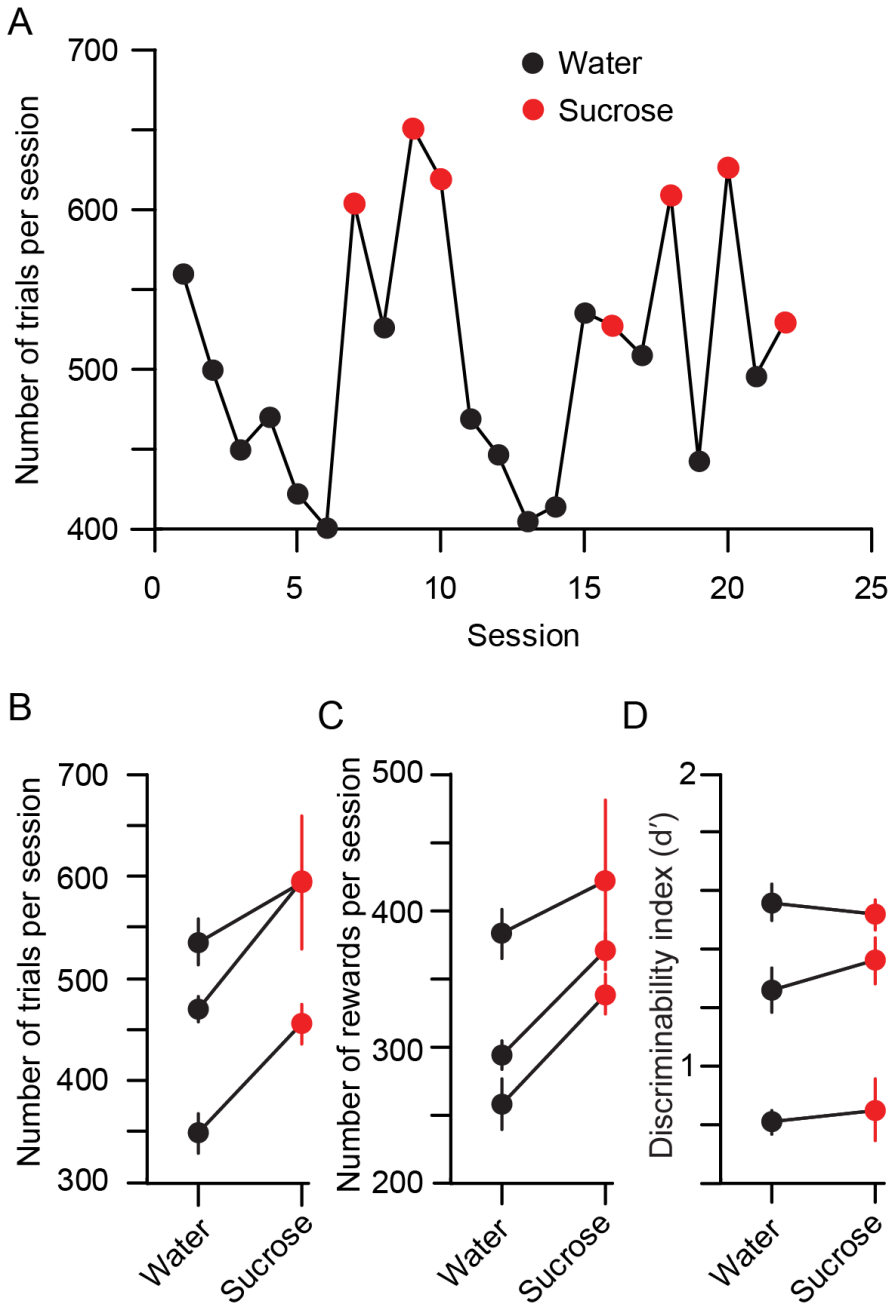
Supplementing water rewards with sucrose increases the number of trials performed by mice. A. Example experiment, with water (black circles) and sucrose (red circles) rewards provided on alternating sessions. B. The number of trials is 23% larger with sucrose (p<0.001 in two mice; n.s. in the third). C. The number of rewards per session is larger (p<0.001 in two mice; n.s. in the third). D. The discriminability index is unchanged.

The lick-left/lick-right object location discrimination task described above has a delay epoch to separate sensation and action, enabling study of perceptual decision. It usually takes 3–6 weeks to train mice to perform this task using a single (C2) whisker. Higher performance and shorter training times can be achieved if either the delay epoch is removed or mice are allowed to perform the task with multiple whiskers [46]. We often use a modified lick-left/lick-right object location discrimination task without delay (data in **Figure 13**). This task does not have a delay epoch, and mice perform object location discrimination with a row of whiskers. In addition, there were eight possible pole positions (evenly spaced at 1 mm) on the right side of the face (5 mm lateral to the whisker pad). The pole positions were held constant from session to session. Mice were trained to indicate the four posterior pole positions with licking right, and the four anterior pole positions with licking left.

The lick-left/lick-right task with a delay epoch was also trained using an alternative strategy that used a motorized lickport. The left and right lickports were mounted on a stepper motor (Zaber Technologies, P/N NA08B30) which was controlled by a computer (i.e. the motorized lickport). The lickport was positioned so that it was centered along the animal's medial-lateral axis, but rested approximately 5 mm out of reach of the tongue. Immediately before the response epoch, the lickport was quickly moved within reach of the tongue (0.25 s) and mice initiated licking. Upon reward collection, or immediately after an incorrect response, the lickport was withdrawn. Most mice learn to withhold licking until the lickport moves into reach. This version of the task does not have a punitive stimulus (sound or timeout) to train a delay.

### 7. Sucrose rewards

To motivate mice to consume more water and thus perform more trials, we supplemented sucrose in water at 0.1 g/ml concentration (50 g sucrose and 1.7 g cool-aid black cherry mixed with water to 500 ml final volume). We trained three mice to perform the modified lick-left/lick-right object location discrimination task. Water or sucrose water was used on alternating sessions (**Figure 13A**). The reward liquid drop size was kept constant at 4 µL. The behavioral session was terminated when the mouse showed signs of being sated (e.g. reduced whisking, occurrence of trials without licking response). Mice were supplemented to 1 ml if they drank less than that amount in any behavioral session. This is to prevent mice from being thirstier on the subsequent session. Mice performed a significantly higher number of trials and obtained more rewards in sucrose water sessions (**Figure 13B, C**). The performance using sucrose water was not increased (**Figure 13D**). To assess potential adaption to sucrose reward, after one month of interleaved testing, we tested sucrose reward for an additional 15 consecutive sessions. Mice consistently consumed more sucrose reward compared with water. The caloric intake from sucrose is about 5% of total caloric intake in a normal mouse (http://www.jax.org/phenome). We did not observe obesity in mice trained on sucrose water for up to four months. Thus sucrose water boosted the number of trials per session without compromising the animals' performance and health.

## Discussion

We describe procedures for training head-fixed mice to perform robust perceptual behaviors. In each trial mice were exposed to one of several sensory stimuli and had to choose one of two responses based on the sensory stimuli. The behavioral choice was signaled by mice touching a water port with their tongue. Mice were water restricted, and thus motivated by thirst. Mice performed many hundreds of behavioral trials per session for water rewards. Weight loss associated with water restriction was positively correlated with the animals' behavioral performance and the number of correct trials (**Figure 5A, B**). Trained mice consumed 1 ml water per day during behavioral sessions. Mice maintained good health for four months of continuous water restriction (**Figure 12**).

The water restriction procedure was developed for C57BL/6J mice and worked for all inbred laboratory strains we have used (C57BL/6Crl, PV-IRES-Cre, Six3-Cre, Scnn1a-Tg3-Cre, VGAT-ChR2-EYFP) [7], [17]–[23]. Water restriction has to be adjusted depending on the relative humidity. Many species of mice survive, and even maintain their weight, without access to water at moderate levels of humidity [42]. Mice can derive their entire fluid intake from moist food. Laboratory mouse strains can vary with respect to their water consumption by several-fold (http://www.jax.org/phenome). The water schedule may also have to be adjusted according to mouse strain and sex. Furthermore, water restriction schedules also have to take activity in the home cage into account. Mice housed in enriched environments with access to treadmills need more water.

Our studies have focused on active tactile sensation in the sense that mice have to move their whiskers to accumulate information about tactile stimuli. Although it has long been appreciated that natural sensation is active [27], [47]–[50], neurophysiological studies of perception usually probe situations in which stimuli are applied passively (i.e. in fixating or immobilized non-human primates) [51], [52]. In our behaviors mice controlled the position of the whiskers (but not their head) and thus the sensory input. Head-fixation was critical for these experiments because it facilitates precise measurements of the dynamics of whiskers and their interactions with objects [17], [18], [30].

Mice were trained on either a lick/no-lick (go/no-go) or a lick-left/lick-right object location discrimination task. The lick/no-lick task has been successfully used to study neuronal correlates of perception [18], [22], sensorimotor integration and learning [19]–[21]. The lick/no-lick task has some disadvantages for the study of perceptual decisions. First, mice are intrinsically biased towards licking; that is, animals usually prefer licking to get water reward in “go” than withholding licking to avoid timeouts in “no-go” trials. This complicates the interpretation of psychometric curves and perturbation experiments [8], [18]. Second, after a few touches with the pole, mice initiate licking within 100's of ms.. Thus the sensation of touch and action (i.e. licking) happen nearly simultaneously. To delineate “sensation” and “action” in time, we developed the lick-left/lick-right object location discrimination task with a delay epoch [7]. Mice accumulated tactile information during the sample epoch and maintained a memory of pole location or motor choice during the delay epoch. Though the lick-left/lick-right task has the advantage of separating behavioral events (e.g. whisker touch and licking) in time, it typically requires additional training time. In addition, the lick/no-lick task has trials without reward and licking, which can be helpful to isolate neural activity related to specific behavioral variables. We have also noticed differences in whisking strategies across the two types of behavioral tasks [7], [18].

The lickport position plays a crucial role in training. In the lick/no-lick task, if the lickport is too close mice tend to lick compulsively irrespective of trial type. If the lickport is too far, mice will tend to miss rewards and become discouraged. Adjusting the lickport position for individual mice is critical in behavioral shaping. In the lick-left/lick-right task, the left and right lickports are usually placed symmetrically along the midline of the animal's mouth. However, some mice have intrinsic licking bias and prefer to lick to one side over the other. This intrinsic bias can be countered by moving the preferred lickport laterally away from the animal's mouth. We ensured that the lickport positions are unchanged between experimental sessions, with occasional modifications to counteract animals' bias.

Although we focus our description on training active tactile behaviors, the core components of the methods can be used to train mice on other perceptual tasks. Training was divided into multiple stages (e.g. **Figure 12**). These stages can be grouped as follows: learning the mechanics of water rewards; learning trial and reward timing; associating reward with a stimulus (sometimes this stage was combined with the previous stage); when appropriate, learning about delays between stimulus and reward; learning perceptually more difficulty discriminations; reversal of stimulus – reward contingency (not discussed here). Mice were advanced from easier tasks to the next level when they performed at 70% correct. Mice were advanced promptly to avoid habit formation.
