# Correction: Procedures for Behavioral Experiments in Head-Fixed Mice

**DOI:** 10.1371/journal.pone.0101397

**Published:** 2014-06-26

**Authors:** The PLOS ONE Staff

There is an error in Figure 2. Please see the corrected Figure 2 here.

**Figure 2:**
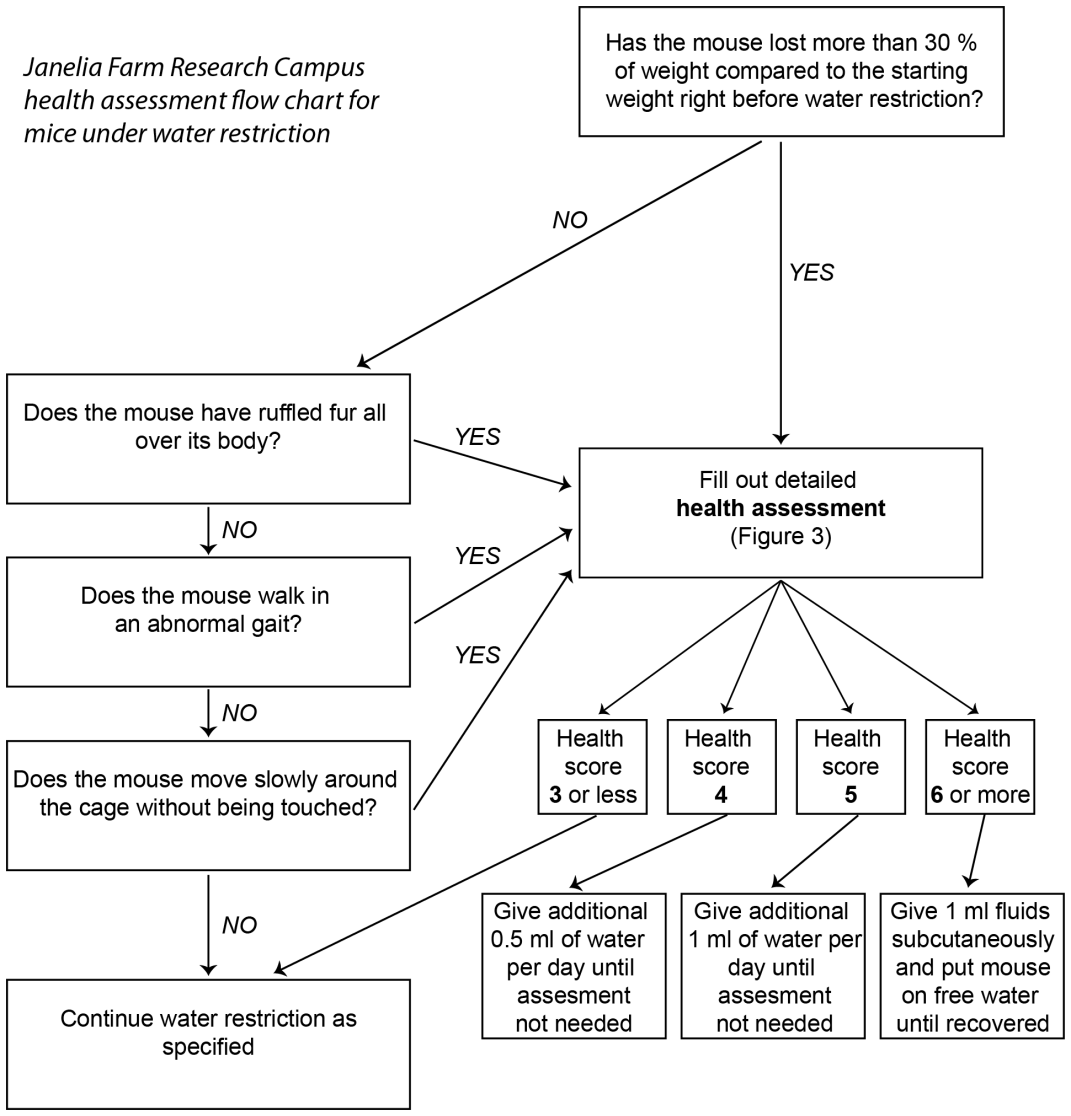
Flowchart for monitoring mice under water restriction.
